# Supplementary material for: From sequence to enzyme mechanism using multi-label machine learning
Source: BMC Bioinformatics. 2014 May 19;15:150. doi: 10.1186/1471-2105-15-150 (PMC4229970; doi:10.1186/1471-2105-15-150)
Supplement: Additional file 2 — Java code of ml2db. Additional file ml2db_code.tar.gz contains the Java source code to run the multi-label machine learning experiments and save the results to database. The code’s Javadoc is included. [file 1471-2105-15-150-S2.zip › additional file 2/ml2db/ml2db/doc/main/ml2db/weka/JaccardDistanceTest.html]

JaccardDistanceTest


JavaScript is disabled on your browser.


- Overview
- Package
- Class
- Use
- Tree
- Deprecated
- Index
- Help

- Prev Class
- Next Class

- Frames
- No Frames

- All Classes

- Summary:
- Nested |
- Field |
- Constr |
- Method

- Detail:
- Field |
- Constr |
- Method


main.ml2db.weka

## Class JaccardDistanceTest

- java.lang.Object
- - main.ml2db.weka.JaccardDistanceTest

- ---

    

  ```
  public class JaccardDistanceTest
  extends java.lang.Object
  ```

  Version:
  :   21 Apr 2013

  Author:
  :   ldf

- - ### Constructor Summary

    Constructors

    Constructor and Description

    `JaccardDistanceTest()`
  - ### Method Summary

    Methods

    | Modifier and Type | Method and Description |
    | --- | --- |
    | `static JaccardDistanceStored` | `getJaccardDistanceTestSet()` |
    | `static void` | `printDist(weka.core.Instance a, weka.core.Instance b, double dist)` |
    | `void` | `setUp()` |
    | `void` | `testDistanceInstanceInstance()` Test method for `JaccardDistanceStored.distance(weka.core.Instance, weka.core.Instance)` . |

    - ### Methods inherited from class java.lang.Object

      `equals, getClass, hashCode, notify, notifyAll, toString, wait, wait, wait`

- - ### Constructor Detail


    - #### JaccardDistanceTest

      ```
      public JaccardDistanceTest()
      ```
  - ### Method Detail


    - #### setUp

      ```
      public void setUp()
                 throws java.lang.Exception
      ```

      Throws:
      :   `java.lang.Exception`


    - #### testDistanceInstanceInstance

      ```
      public void testDistanceInstanceInstance()
      ```

      Test method for
      `JaccardDistanceStored.distance(weka.core.Instance, weka.core.Instance)`
      .


    - #### getJaccardDistanceTestSet

      ```
      public static final JaccardDistanceStored getJaccardDistanceTestSet()
      ```


    - #### printDist

      ```
      public static void printDist(weka.core.Instance a,
                   weka.core.Instance b,
                   double dist)
      ```


- Overview
- Package
- Class
- Use
- Tree
- Deprecated
- Index
- Help

- Prev Class
- Next Class

- Frames
- No Frames

- All Classes

- Summary:
- Nested |
- Field |
- Constr |
- Method

- Detail:
- Field |
- Constr |
- Method
